# Bayesian evidence synthesis to estimate HIV prevalence in men who have sex with men in Poland at the end of 2009

**DOI:** 10.1017/S0950268815002538

**Published:** 2015-11-06

**Authors:** M. ROSINSKA, P. GWIAZDA, D. DE ANGELIS, A. M. PRESANIS

**Affiliations:** 1 Department of Epidemiology, National Institute of Public Health – National Institute of Hygiene, Warsaw, Poland; 2 Department of Mathematics, Informatics and Mechanics, University of Warsaw, Warsaw, Poland; 3 Medical Research Council Biostatistics Unit, Cambridge, UK

**Keywords:** Bayesian analysis, HIV, men who have sex with men, Poland

## Abstract

HIV spread in men who have sex with men (MSM) is an increasing problem in Poland. Despite the existence of a surveillance system, there is no direct evidence to allow estimation of HIV prevalence and the proportion undiagnosed in MSM. We extracted data on HIV and the MSM population in Poland, including case-based surveillance data, diagnostic testing prevalence data and behavioural data relating to self-reported prior diagnosis, stratified by age (⩽35, >35 years) and region (Mazowieckie including the capital city of Warsaw; other regions). They were integrated into one model based on a Bayesian evidence synthesis approach. The posterior distributions for HIV prevalence and the undiagnosed fraction were estimated by Markov Chain Monte Carlo methods. To improve the model fit we repeated the analysis, introducing bias parameters to account for potential lack of representativeness in data. By placing additional constraints on bias parameters we obtained precisely identified estimates. This family of models indicates a high undiagnosed fraction [68·3%, 95% credibility interval (CrI) 53·9–76·1] and overall low prevalence (2·3%, 95% CrI 1·4–4·1) of HIV in MSM. Additional data are necessary in order to produce more robust epidemiological estimates. More effort is urgently needed to ensure timely diagnosis of HIV in Poland.

## INTRODUCTION

Men who have sex with men (MSM) are at present one of the groups most vulnerable to experiencing HIV outbreaks. After a decline of drug-injection-related epidemics in Europe and North America as a consequence of implementing intense harm-reduction measures, many countries are now seeing a re-emergence of sexually transmitted HIV, especially in MSM [1, 2].

There is also growing evidence that HIV has become an important public health issue in MSM in Poland [3]. An increase in the number of new diagnoses in this group was observed after 2005–2006, but it is not clear to what extent this tendency may be related to wider testing of a largely undiagnosed pool of infected MSM and to what extent it represents a trend in newly acquired infections. In many countries, the MSM population is targeted for extensive screening [4] and new technologies are being investigated to further scale up screening [5–8]. High coverage of such screening is necessary to fully benefit from potential incidence reduction due to early treatment [9], as undiagnosed infections pose a serious public health concern. First, they can contribute to increased morbidity and treatment costs of advanced-stage infections. It has been estimated that late presentation, especially with initial CD4 counts <100 cells/mm^3^, causes a substantial increase in direct medical costs of HIV care [10, 11]. Second, undiagnosed infections may drive the (re-)emergence of the HIV epidemic. Understanding the extent of HIV spread, the prevalence, as well as the proportion of infected people who are undiagnosed, may help inform the planning and evaluation of testing strategies, as well as the prediction of the future burden of disease and consequent treatment needs.

Although HIV testing is promoted in MSM in Poland, the proportion of infections that are undiagnosed remains largely unknown. Despite clear evidence of HIV spread in MSM in Poland during recent years, the most recent seroprevalence study was conducted in 2004, in 12 cities in Poland, revealing a prevalence of 4·7%; about 40% of infections in the study were still undiagnosed [12]. Since then, estimation of HIV prevalence in this group in Poland has not been attempted. Although several data sources exist, they do not allow direct estimation of either the prevalence or the diagnosed fraction. They include diagnostic testing data, self-reported behavioural data and data originating from case-based surveillance of diagnosed infections. Moreover, the quality of the information provided remains problematic. The surveillance data suffer from underreporting of the exposure category and possible misclassification of MSM to other exposure categories, due to reservations in disclosing MSM status [3]. The survey data, on the other hand, are often based on convenience sampling or rely on volunteers. Although sampling methodology for hard-to-reach populations, such as MSM, has recently improved, many challenges still remain [13]. In consequence, it is often difficult to determine if the study sample is representative for the overall population of MSM, as well as to understand potential biases. These challenges preclude the use of direct estimation methods [14]. However, it is still possible to estimate the main parameters of interest, such as HIV prevalence, by combining imperfect and indirectly related data sources. Understanding how these data are collected allows explicit definition of the quantities measured by them in terms of functions of the main parameters of interest, such as prevalence and the undiagnosed fraction. All data sources may be then combined into one stochastic model described in terms of these main parameters (‘evidence synthesis’). Importantly, this indirect method offers also the possibility of detecting conflicts between data sources and adjusting for possible biases [15].

The aim of this study is to combine existing evidence to estimate HIV prevalence and the proportion of infections that are undiagnosed in MSM, possibly adjusting for biases in different data sources.

## METHODS

### Bayesian evidence synthesis

Bayesian multi-parameter evidence synthesis (e.g. [16–19]) was chosen as a flexible tool to combine available data and prior knowledge. This method, as applied to HIV, was first developed in the UK and then successfully implemented in other countries [20–22]. We note that in Poland the data sources are not as abundant as in the previously mentioned examples, and thus a frequentist evidence synthesis method could be also considered. However, selecting a Bayesian approach offers greater flexibility to expand the model should other data become available, as well as to explicitly introduce expert opinion [23]. The Bayesian framework consists of defining a *prior* distribution, *f*(*θ*), describing what is known about the parameters *θ* of a model before seeing the data **Y**; updating the prior with the *likelihood* 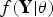 of the data given the parameters; and therefore obtaining a *posterior* distribution, 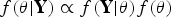, for the parameters that synthesize the prior knowledge with the observed data [24]. In our model, we consider the basic parameters of interest to be: the proportion of MSM in males in Poland (*ν*); prevalence of HIV (*π*); and the fraction (*δ*) of HIV infections in MSM in Poland that are diagnosed. Knowing these quantities, we are able to estimate the number of MSM who are diagnosed with HIV (*Nνπδ*, where *N* is the total number of men in Poland), as well as the number of those who are infected but are, as yet, undiagnosed (*Nνπ*(1 − *δ*)). Although direct information on the desired parameters in Poland does not exist, there are numerous data items which can be described as realizations of distributions determined by parameters that are functions of the basic parameters. For example, the frequency of positive results in diagnostic testing data may be thought to represent the prevalence of previously undiagnosed infection and hence be expressed in terms of prevalence and the undiagnosed fraction: *π* × (1 − *δ*). Full model details are given in the ‘Initial model’ section below.

The joint posterior distribution for the basic parameters and their marginal distributions are estimated using a Markov Chain Monte Carlo (MCMC) algorithm in OpenBUGS [25]. The convergence of the algorithm was inspected visually and with the Brooks–Gelman–Rubin statistic. Median values and 95% credibility intervals (CrI) from the posterior distribution are presented.

#### Model criticism

With the complexity of the models considered, assessment of model fit and choosing between competing models is critical. We used deviance summaries to assess and compare models [26, 27]. The contribution to posterior expected deviance of each data point is compared to 1 to explore the goodness of fit of a specific model to the data. To discriminate between different models, we used the Deviance Information Criterion (DIC). The DIC is analogous to Akaike's Information Criterion, used in frequentist analysis, in that it combines a measure of goodness of fit, posterior mean deviance, with a penalty for the model complexity in the form of the effective number of parameters. To compare models including partially different datasets we used summed contributions to the DIC for the common data points. Further details of methods are given in the Supplementary material.

### Data sources and their limitations

#### Case-based reporting of diagnosed cases

Case reporting was implemented in 1986 (the first cases diagnosed in Poland in 1985 were included). It is based mainly on reports from laboratories performing confirmatory assays, although clinicians are also mandated to report cases that they diagnose. HIV diagnosis can be made through voluntary testing, either at counselling sites (often not involving clinicians) or directly at private laboratories (upon request of an individual). These cases might be reported by clinicians only when they present for care. Important information such as transmission category or clinical status at diagnosis is often missing for new HIV diagnoses reported only by laboratories. The EU case definition is followed, which includes only confirmed HIV diagnoses. A name-based identifier is used to exclude duplicates at regional and national levels. However, this identifier may be deleted from the report by request of the HIV-infected person. Deaths of persons who develop AIDS are also reportable, although death reporting may be incomplete. The central registry is maintained at the Department of Epidemiology of the National Institute of Public Health, National Institute of Hygiene. For this study, we extracted data on all cases diagnosed by 31 December 2009, reported by 30 June 2013 and not known to be dead by 31 December 2009. We restricted the data set to male cases only, including those reported as MSM and those with transmission category missing.

#### Data from voluntary counselling and testing *(*VCT*)* sites

The VCT network provides a sentinel system for characterization of testing patterns and frequency of new diagnoses in testers. There were 30 centres included in the network in 2010 in major cities in Poland, located in different institutions, including medical facilities, public health authorities and Non-Governmental Organizations. To ensure quality of service and data collection, the consultants are trained to follow the same procedure by the National AIDS Centre. VCT sites offer free-of-charge testing accompanied by pre- and post-test consultation with a trained advisor. The sites are accessible to everyone and do not specifically encourage any particular demographic group or HIV-affected subpopulation to come forward for testing, although testing based on higher levels of risky behaviours is promoted. At presentation for testing, a consultant performs individual risk assessment for each person requesting an HIV test, and completes a structured questionnaire, which is then forwarded to the National AIDS Centre for yearly processing. The questionnaire includes basic demographic and risk information, as well as HIV test results. The questionnaire is anonymous and possible duplicates are not excluded. A dataset of all MSM attending any of the VCT sites in 2010 was made available by the National AIDS Centre. This dataset was restricted to exclude records of clients who were not tested, those previously known to be HIV positive and those of clients who reported having already attended a VCT site in 2010 to avoid double counting. Although data on all clients of the VCT network were included in the analysis, the representativeness of this group for the total population of MSM in Poland could be questioned. First, the VCT sites are located in large cities and they reach a predominantly urban population. Travel distance may be a barrier for smaller cities and villages. Second, the VCT sites rely on volunteers who self-refer for testing. This population may differ in terms of risk behaviours and socioeconomic characteristics, e.g. educational status, from the general population of MSM. The network is, in fact, promoted especially to attract persons with higher levels of risk behaviours (The National HIV/AIDS Programme, 2012–2016).

#### Behavioural and demographic data

Summary data by age and region from an internet behavioural survey (the European MSM Internet Survey; EMIS) were available from the National AIDS Centre. EMIS is a self-reported online survey run simultaneously in 25 languages in Europe in June–August 2010. Potential respondents were invited through individual messages from online social networks or through banners on (country-specific) websites (www.emis-project.eu). Additional information can be found in [28]. EMIS was based on a convenience sample and consequently could be prone to biases. By comparison to other data sources, biases have been identified with respect to age and frequency of HIV diagnoses [29].

Additionally, data from a household survey, with a self-administered questionnaire on sexual behaviours, were used to inform the prevalence of MSM (any sexual contact with another man in his lifetime) in the general population. The survey was implemented in 2001 among persons aged 15–49 years [30].

The source population size was extracted from census data available at www.stat.gov.pl.

### Initial model

The different data sources may overlap in terms of the individuals included: the new diagnoses established in VCT in 2010 may also be reported to surveillance, i.e. may appear in the cumulative number diagnosed to end of 2010. Individuals participating in EMIS in 2010 may also appear either in the VCT or surveillance data. However, it is not possible to identify the overlaps between sources, and hence to model them dependently. To proceed, we therefore assumed independence between data sources, while aiming to minimize the overlaps, by carrying out estimation for the end of 2009, in men aged ⩾18 years. First, we use the cumulative diagnoses observed in surveillance up to the end of 2009 only, excluding diagnoses made in 2010. Second, we use the VCT and EMIS data in 2010 to represent, respectively, undiagnosed and diagnosed prevalence at the end of 2009, as explained in the following section.

We subdivided the population into the capital city of Warsaw and surroundings (approximated by the Mazowieckie region, designated ‘Maz’) and the rest of Poland (designated ‘Oth’). This categorization may be especially relevant for HIV, as over the course of the past decade, the number of new HIV diagnoses displayed the greatest dynamics in the Mazowieckie region [3]. We also included stratification into younger (years ⩽ 35) and older (>35 years) men.

Consequently, we defined nine basic parameters: *π*_age,reg_, the HIV prevalence by age group and region; *δ*_age,reg_, the proportion diagnosed by age group and region; and *ν*, the proportion of MSM in the general male population, assumed to be the same across age groups and regions. Each of these parameters were given independent flat prior distributions giving equal probability to each value in [0, 1], i.e. 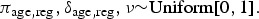


#### Likelihood contributions

Data sources informing particular parameters are presented in Table 1 and are described below.

**Table 1.** Data directly and indirectly informing modelled parameters

Definition	Basic or functional parameter	Data	Source
Proportion of MSM in men aged ⩾15 years	*ν*	35/1536	General population survey
Number of diagnosed HIV cases in MSM (by age, region)	*d*_age,reg_ = *N*_age,reg_ · *ν* · *π*_age,reg_ · *δ*_age,reg_		
Number of reported HIV cases in MSM (by age, region)	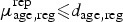		Case-based surveillance
	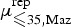	77	
	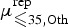	198	
	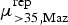	163	
	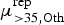	398	
Number of reported HIV cases in MSM or men with unknown exposure category (by age, region)	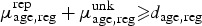		Case-based surveillance
	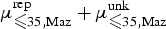	325	
	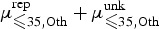	1670	
	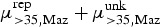	564	
	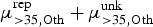	2475	
Prevalence of diagnosed infection (by age, region)	*dp*_age,reg_ = *π*_age,reg_ · *δ*_age,reg_		EMIS survey
	*dp*_⩽35,Maz_	31/776	
	*dp*_⩽35,Oth_	44/1549	
	*dp*_>35,Maz_	17/188	
	*dp*_>35,Oth_	21/327	
Prevalence of undiagnosed infection (by age, region)	*u*_age,reg_ = *π*_age,reg_ · (1 − *δ*_age,reg_)		VCT data
	*u*_⩽35,Maz_	41/1053	
	*u*_⩽35,Oth_	49/1272	
	*u*_>35,Maz_	18/183	
	*u*_>35,Oth_	28/217	
Past 6-month testing rate in previously undiagnosed (by age, region)	*TR*_⩽35,Maz_	216/739	EMIS survey
	*TR*_⩽35,Oth_	313/1488	
	*TR*_>35,Maz_	28/169	
	*TR*_>35,Oth_	51/298	
Prevalence in tested past 6 months, not diagnosed >6 months prior to the survey (by age, region)	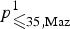	12/216	EMIS survey
	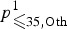	4/313	
	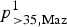	1/28	
	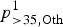	2/51	

EMIS, European MSM Internet Survey; VCT, voluntary counselling and testing.

#### Undiagnosed prevalence by age and region

The VCT (testing) data represent aggregated values over a period of time of a single year, 2010. The percent of tests resulting in new diagnoses carries information about the prevalence of previously undiagnosed infections, which is possibly changing over the course of this one year, depending on the rate of diagnoses as well as the rate of new infections. To understand the diagnosis process and establish an appropriate model for the data, we consider the following assumptions. First, we assume that an individual *i* coming for a test on the date *t* has a test result *y*_*i*_ that can be viewed as a random draw from the Bernoulli distribution, with some unknown parameter *u*(*t*), dropping temporarily the age and region indices for ease of presentation. We note that since the VCT data do not include people who were known to be previously diagnosed, *u*(*t*) does not exactly represent undiagnosed prevalence *π*(*t*)(1 − *δ*(*t*)), for which the denominator population is all MSM, including those previously diagnosed. However, since the number diagnosed represents a small fraction of the total population, we will assume that *u*(*t*) is sufficiently close to the true undiagnosed prevalence and we will not make the distinction between these two quantities i.e. 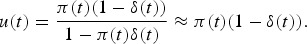 Next, we assume that over the course of one year the rate of diagnoses in the population *r*_d_ and the rate of new infections in the population *r*_i_ remains constant. We also assume that the overall population of MSM is stable over the course of the year, i.e. that entries and exits to the population (due to aging, migration, death and behaviour change) cancel. We may then write: 1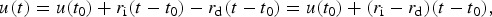 where *t* ∈ [*t*_0_, *t*_1_], with *t*_0_ denoting 1 January 2010 and *t*_1_ denoting 31 December 2010.

We note that due to equation (1), if the number of diagnoses corresponds to the number of new infections, then *u*(*t*) will not change over time. If this were the case, a simplifying assumption could be made that the VCT data may inform *u*(*t*) at any time point in 2010, including at the start of the year or end of 2009. In order to challenge this assumption for the interval [*t*_0_, *t*_1_], we run a binomial regression model on the VCT data, with outcome the HIV-positive result and test date as the explanatory variable. No significant trend was noted and the details of this analysis are presented in the Supplementary material (Fig. S1, Table S1). We therefore concluded that during the year 2010 the undiagnosed prevalence remained approximately the same and the VCT data can be used to inform the undiagnosed prevalence at the end of 2009, *u*(*t*) = *u*(*t*_0_) = *u*. We may therefore specify the contribution of the VCT data to the likelihood as a series of Bernoulli trials for each individual with parameter *u*, or equivalently, the summed positive test results (Table 1) 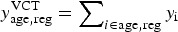 being drawn from a Binomial distribution: 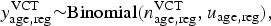


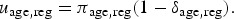


*Diagnosed prevalence by age and region.* The number of EMIS respondents self-reporting a diagnosis prior to the end of 2009, 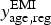 (Table 1), was assumed to be a draw from a Binomial random variable, with denominator the total MSM respondants who answered the question, 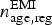 and probability parameter the (previously) diagnosed prevalence *dp*_age,reg_: 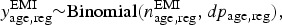


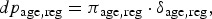


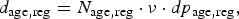
 where *d*_age,reg_ denotes the total number of diagnosed cases by age and region and *N*_age,reg_ is the total population of men aged >18 years by age group and region: age = ⩽35, >35; reg = Maz, Oth. The parameter *d*_age,reg_ is additionally informed by the number of registered cases in the surveillance data by age and region. However, due to a large proportion of missing information on the risk group in the surveillance data, we were not able to use the registered numbers directly. We assumed that the true number of diagnoses lies between the reported number of HIV cases among MSM and the number of male cases reported either as MSM or with missing transmission category, i.e. 2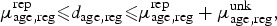 and the observed counts 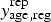 and 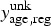 are modelled as realizations of Poisson distributions with means of 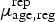 and 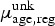, respectively.

*MSM in the general population.* The proportion of men in the general population, *n*^pop^, who are MSM, *y*^pop^ (Table 1), is taken to be a realization of a Binomial random variable: 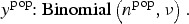


We also define the overall prevalence and overall diagnosed fraction: 
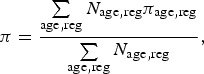


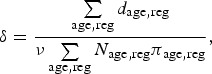


**Fig. 1.**
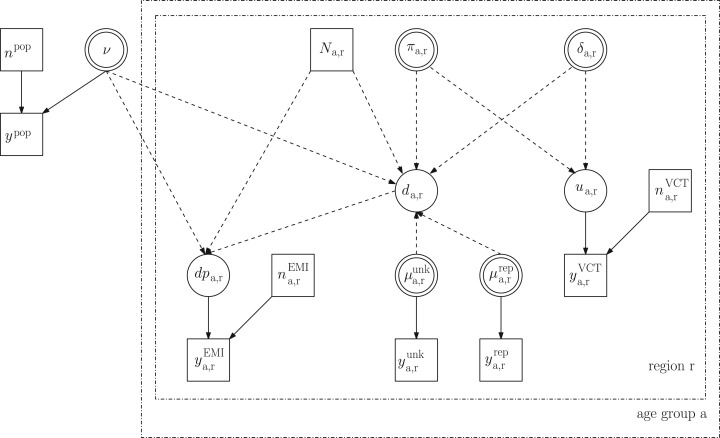
Directed acyclic graph of the initial model (see description in the text).

Figure 1 is a directed acyclic graph (DAG) visualizing the relationships between the data and the parameters in the model. The square nodes represent observed data; the double circle nodes are parameters for which a prior distribution is assumed; and the single circle nodes are the parameters defined as functions of other parameters. Further, solid arrows denote stochastic relationships, i.e. the parent nodes determine the distributions of the child nodes. The dashed arrows specify deterministic functional relationships, i.e. that the child node can be expressed as a function of the parent nodes. The dash-dotted rectangles represent repetition, so that the relationships inside the inner rectangle are specified separately in each age/region stratum.

## RESULTS

### Initial model

Initial model M0 assumes that all data sources provide unbiased samples from the total population of MSM in Poland. The summary results of the model are presented in Table 2. Analysing the model fit, the deviances for the majority of the data points fall close to unity, except notably the deviance for data corresponding to the proportion of MSM in the general population of men, *ν* (Table 2). This observation indicates potential conflict between the results of this general population survey and the other data points. Compared to the observed percentage 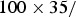 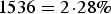, the estimate for *ν* in this model was approximately 0·8% (95% CrI 0·5–1·0) (Table 2), which also stands in conflict with typical estimates observed in many countries, e.g. [31]. This suggests that some of the other data may be biased. In addition, we note the large deviance value for the EMIS data, suggesting that some lack of fit may in fact concern this dataset.

**Table 2.** Comparison of basic parameters (median, 95% credible interval) and deviances between the considered models

	Estimates under different models							
Parameter	M0	M1	M2	M3	M4	M5	M6	M7
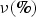	0·8 (0·5–1)	2·1 (1·4–2·8)	2·1 (1·5–2·9)	2·1 (1·4–2·8)	2·1 (1·4–2·8)	2·1 (1·4–2·8)	2 (1·4–2·8)	2·3 (1·6–3·1)
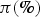	12 (9·7–14·8)	9·9 (7·6–12·5)	6·2 (1·6–11)	2·3 (1·4–3·9)	2·4 (1·4–4·1)	2·8 (1·3–8·3)	3·2 (1·3–9·7)	1·4 (0·9–2·4)
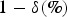	76·6 (69·6–82·3)	93·1 (88·9–95·9)	88·9 (58·8–95)	68·3 (59·3–76·1)	69·1 (57·4–79·2)	74·3 (54·2–91·7)	77 (54·2–93·4)	60 (51·2–69)
Number diagnosed	3353 (2788–3843)	2228 (1513–2800)	2206 (1495–2783)	2394 (1764–2887)	2364 (1680–2874)	2298 (1566–2844)	2284 (1550–2838)	2071 (1424–2654)
Number undiagnosed	10 970 (7099–16 310)	29 610 (18 890–44 880)	17 550 (3093–37 530)	5063 (3186–8124)	5212 (2921–9230)	2547 (6605–24 420)	7564 (2551–30 000)	3033 (1935–5099)
Bias estimates								
Bias in EMIS data								
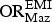	—	5·4 (2·7–9·8)	5·5 (2·8–9·9)	6·5 (3·8–10·6)	6·2 (3·6–10·4)	5·7 (3·0–10·0)	5·6 (2·9–9·9)	7·1 (3·7–12·5)
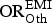	—	8·6 (4·4–15·8)	8·8 (4·5–16·1)	6·5 (3·8–10·6)	6·9 (3·9–12·1)	7·6 (4·0–14·4)	7·8 (4·1–14·8)	11·6 (6·1–20·1)
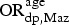	—	1·9 (1–3)	1·9 (1·1–3)	2·0 (1·2–3·2)	2·0 (1·2–3·1)	2·0 (1·1–3·1)	1·9 (1·1–3·1)	2·2 (1·5–3·3)
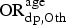	—	2 (1·2–3·2)	2 (1·2–3·3)	1·8 (1·1–2·8)	1·8 (1·1–3)	1·9 (1·1–3·1)	2·0 (1·1–3·2)	3·0 (2·1–4·3)
Bias in VCT data								
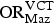	—	—	1·5 (1–13·1)	6·5 (3·8–10·6)	6·3 (3·4–11·8)	4·7 (1·2–13·8)	3·9 (1–13·6)	7·1 (3·7–12·5)
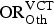	—	—	1·7 (1–24)	6·5 (3·8–10·6)	6·3 (3·4–11·9)	5·0 (1·2–15·8)	4·4 (1–17·4)	11·6 (6·1–20·1)
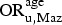	—	—	2·7 (1·5–4·7)	2·7 (1·5–4·7)	2·7 (1·5–4·7)	2·7 (1·5–4·7)	2·7 (1·5–4·7)	2·2 (1·5–3·3)
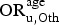	—	—	3·7 (2·2–6·0)	3·7 (2·2–6·0)	3·7 (2·2–6·0)	3·7 (2·2–6·0)	3·7 (2·2–5·9)	3 (2·1–4·3)
Posterior deviance contributions								
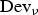	31·17	1·38	1·29	1·39	1·42	1·43	1·43	0·98
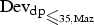	0·92	0·99	0·98	1·02	0·99	0·97	0·98	1·11
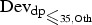	0·89	0·99	0·98	0·99	0·97	0·97	0·97	1·02
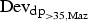	6·31	1·33	1·32	0·80	0·85	1·13	1·20	1·05
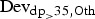	16·22	1·19	1·17	1·95	1·76	1·46	1·41	0·99
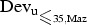	0·99	1·00	0·99	0·99	0·98	0·99	0·99	0·91
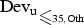	0·99	1·00	0·99	0·98	0·98	0·99	0·99	2·08
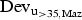	0·98	0·99	0·99	0·98	0·98	0·98	0·99	1·23
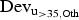	1·00	1·00	1·01	0·98	0·99	0·99	0·99	1·83
DIC	83·5	34·9	34·8	35·0	34·5	34·9	34·9	69·5 (35·9)

EMIS, European MSM Internet Survey; VCT, voluntary counselling and testing; OR, odds ratio; DIC, Deviance Information Criterion.

M0, Initial model; M1, biased EMIS data; M2, both VCT and EMIS data biased; M3, both VCT and EMIS data biased, bias equal; M4–M6, both VCT and EMIS data biased, bias coming from the same distribution with increasing variance (*c* = 0·1, 0·5, 0·8); M7, EMIS nested testing model; Dev_u_, Dev_dp_, for the models accounting for bias, these deviances refer to the biased data.

### Bias modelling

Due to uncertainty regarding the effects of allowing biases in the model, we adopted a stepwise approach to build in parameters for the potential biases in the EMIS and VCT data. We suspect that both studies may include only subsets of the MSM population with increased risk for infection, i.e. that overall prevalence (both diagnosed and undiagnosed) is higher among the study respondents than in the total population of MSM. Furthermore, we also assume an ordered relationship between age-specific prevalences in the source MSM population exists and that despite the biases, this order is maintained in the two study populations. The bias models we implement at each step reflect these prior beliefs.

#### Model M1: bias in the EMIS data only

We denote the diagnosed prevalence in EMIS respondents by 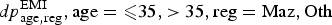. The belief that this diagnosed prevalence is greater than diagnosed prevalence in the general MSM population, *dp*_age,reg_, is expressed via an odds ratio (OR) for being diagnosed with HIV in EMIS respondents relative to the total MSM population, 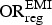: 3
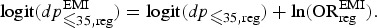


The relationship described by equation (3) is specified for the younger age group, with diagnosed prevalence in the older age group being related to diagnosed prevalence in the younger group via a second bias parameter, 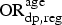: 4
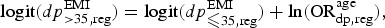

5
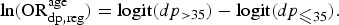


Equation (5) states that the odds ratio 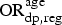 denotes the age-ordered relationship in diagnosed prevalence, not only in the EMIS respondents, but also in the total MSM population, i.e. the true age relation is preserved in the EMIS data.

The odds ratios 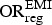 are given a vague prior distribution (on the log scale) admitting only positive values, to reflect the belief of higher prevalence in the EMIS respondents: 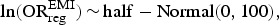 where half-Normal(0,100) signifies the positive half-Normal distribution whose corresponding normal distribution has mean 0 and variance 100.

*Results.* Model M1 has a smaller DIC value than the initial model M0. We note also that the estimated bias parameters are important, with a 95% CrI excluding 1: 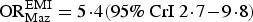, 
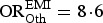
 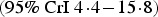. Under this model, the conflict between the number diagnosed and the combination of the proportion diagnosed with the MSM population size is resolved by reducing the proportion diagnosed. This, however, results in an estimated high fraction of infections undiagnosed (93·1%, 95% CrI 88·9–95·9). The high undiagnosed prevalence is informed by the assumed unbiased VCT data. The second-step model (M2) therefore allows the VCT data to be biased as well.

#### Model M2: bias in both the EMIS and VCT data

The bias in the VCT data was modelled similarly to the bias in the EMIS data [equation (3)], assuming a preserved ordered relationship between age groups [equation (5)]. We define the biased prevalences of undiagnosed infection 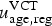, observed in the VCT data, again in terms of two bias parameters per region, 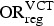 and 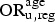. These two parameters represent, respectively: (i) the OR of undiagnosed prevalence in clients of the VCT sites in comparison to the total MSM population, for the younger age group, by region; and (ii) the OR of undiagnosed prevalence in the older age group relative to the younger, again taken to be the same in both the VCT data and in the general population of MSM. We therefore define:
6
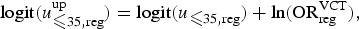

7
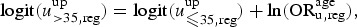

8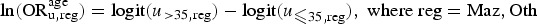 and the following prior distributions for 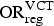, where reg = Maz, Oth: 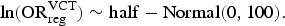


*Results*. The estimated bias parameters for the VCT data in the model M2 have credible intervals above 1, but these are very wide. The DIC, overall deviance and deviance contributions of the particular data points are small, indicating a relatively good fit of the model. However, we note a lack of precision in the estimates for the key parameters, e.g. the estimate of overall prevalence *π* has a 95% CrI of 1·6–11·0 and the corresponding CrI for the undiagnosed fraction (1 − *δ*) is 58·8–95·0. This could be the effect of allowing too much flexibility in the model. The posterior distribution of *π* hints at having two peaks – one for smaller values and another one at approximately 10%.

#### Model M3: constrained biases

In consequence, we attempted to reduce the number of the parameters to be estimated by the model. We did so by introducing a constraint on the biases, considering that both the datasets could be similarily biased. To support this, the estimated biases in the model M2 had largely overlapping credible intervals and the two study groups share similar demographic characteristics (Supplementary Table S1). In model M3 we assumed:
9
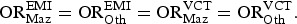


**Fig. 2.**
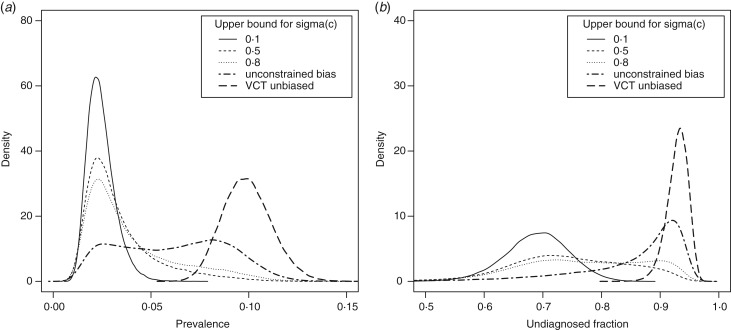
Density plots for overall HIV prevalence and the undiagnosed fraction in models with relaxation of the constraint on bias compared to the density plots for the model with only VCT data biased.

*Results and sensitivity analysis.* The model M3 has acceptable deviance estimates and DIC value similar to the previous models. We obtain also more precise estimates for the overall prevalence and the undiagnosed fraction, which are both in this model lower than in the model allowing for bias in the EMIS data only, with posterior medians of 2·3% *vs.* 9·9% and 68·3% *vs.* 93·1% respectively. We consider that the assumption (9) of the equality of biases is quite strong. In order to investigate the impact of this assumption on the model fit, we ran a sensitivity analysis allowing the biases to come from the same distribution, instead of being equal. In order to ensure that the ORs are positive, we assign prior distributions on the square root scale. We assume: 10
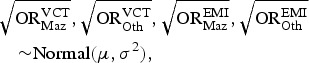
 where *μ* is assigned a flat prior: 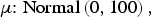


To test the sensitivity of the results to the distribution of the biases, *σ* is assigned a series of priors (models M4, M5, M6): 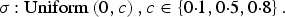


We note that when *c* = 0·1 (model M4), the results are close to the results of model M3 (assuming strict equality of biases). On the other hand, in the model M6 with *c* = 0·8, the constraint is already too weak to effect a visible difference from the unconstrained model M2. Figure 2 shows the changes in the posterior density curves for the overall prevalence *π* and the undiagnosed fraction (1 − *δ*) with increasing flexibility in the bias modelling. Both parameters are well defined for the constrained model, and when the constraint is relaxed, they become diffuse, accommodating larger probability of higher values. We observe that these higher values are consistent with the estimates of the model M1, for which only the EMIS data are considered biased. We conclude that possibly the models M1 and M3 constitute two alternative ways of resolving the conflict in the data, while preserving identifiability of the parameters. Assuming that the VCT data are not likely to be unbiased, we accept model M3 as our final model.

### Model 7: alternative bias modelling using testing process data from EMIS

Taking into account the strong assumptions behind our bias models we additionally explored a different approach to modeling the information available through EMIS to see if the results are consistent. EMIS survey questions allow the testing and diagnosis process in the last 6 months, i.e. end of 2009 up to the study time, to be assessed. Under some assumptions, detailed below, an explicit model of this process may be used to inform not only the diagnosed prevalence, but also the prevalence of undiagnosed HIV infections. First, we will regard the individual records as coming from two time points: *T*_1_, 6 months before participation in the survey; and *T*_2_, the actual date of the survey. We will distinguish between recent (within past 6 months) and long-standing HIV diagnoses, based on the reported date of HIV diagnosis. This distinction will define the status of HIV diagnosis at *T*_1_ (‘known positive’ in case of long-standing HIV diagnosis and ‘no HIV diagnosis’ otherwise). The testing process, which takes place between *T*_1_ and *T*_2_ in those with no HIV diagnosis at *T*_1_ is then captured through recent testing history (past 6 months) and recent HIV diagnosis (past 6 months), in those recently tested.

Let us introduce new parameters referring to the EMIS source population, by age and region:

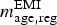
: parameter describing recruitment into EMIS study,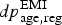: diagnosed prevalence at *T*_1_,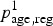: prevalence in those tested between *T*_1_ and *T*_2_,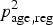: prevalence in those not tested between *T*_1_ and *T*_2_,
*TR*_age,reg_: proportion tested between *T*_1_ and *T*_2_.

Let us consider a nested binomial model to describe the diagnosis process between *T*_1_ and *T*_2_ in the EMIS source population. This model will be informed directly by the data observed in the EMIS survey, assuming that all missing values are missing at random.

We denote the number of individuals who responded to EMIS by *M*_age,reg_, of whom 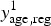 were already diagnosed by *T*_1_, 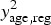 were tested between *T*_1_ and *T*_2_, and 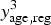 were diagnosed with HIV between *T*_1_ and *T*_2_. Furthermore, 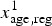, 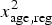 and 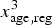 represent the number of observations with missing values at each stage.
11
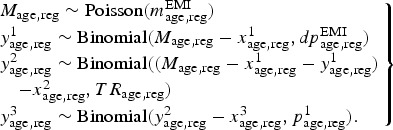


**Fig. 3.**
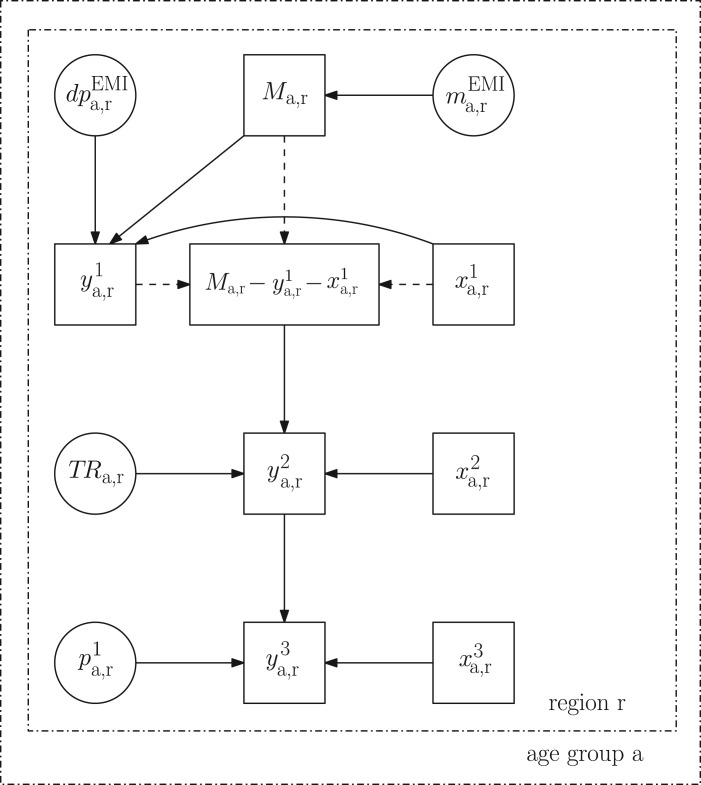
Directed acyclic graph of the nested binomial structure in model M7 (see description in the text).

This nested structure is visualized in the DAG in Figure 3.

To estimate HIV prevalence in those not tested during the preceding 6 months, 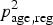, additional assumptions are necessary. As testing of MSM is often driven by risky sexual practices we assumed that the prevelance in those tested is likely higher than in those not tested. Since no other information was available, we assumed that 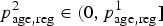.

This assumption leads to the following constraint on the undiagnosed prevalence in the EMIS source population, 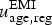, at *T*_1_: 12
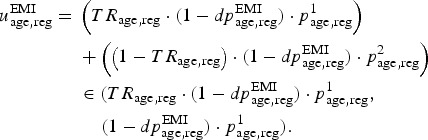


This information is included in the model by setting uniform priors on 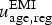 on these intervals. The prevalence 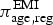 and diagnosed fraction 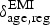 in the EMIS source population at the end of 2009 will be modelled as functions of the parameters introduced above: 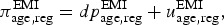


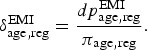


In line with the previously introduced assumptions, we consider the EMIS population to be biased towards a higher risk subgroup, due to the recruitment process. Here, we define prevalence in the general population in terms of prevalence in the EMIS population and a bias parameter, in contrast to the bias models (see ‘Bias modelling’ section above), where we defined the EMIS *diagnosed* prevalence in terms of the general population diagnosed prevalence and a bias parameter. 13
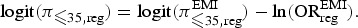


As before, we assume that the age structure, by region, is preserved. 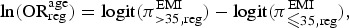


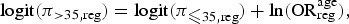


A further assumption is that the diagnosed fraction in the general population is the same as in the EMIS source population, 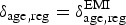. Last, we also assume that the EMIS source population is similar to the VCT source population. This assumption is implemented by: 14
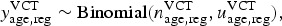

15
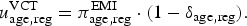


Since the bias parameters for 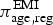 relating them to the underlying parameter *π*_age,reg_ are introduced in multiplicative form [additive on the log scale, equation (13)], by substituting them into equation (15) we see that the same biases will apply to 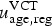 with respect to the true undiagnosed prevalence *u*_age,reg_, i.e. in this model 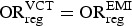. We note further that inclusion of the testing process data available in EMIS, but not in VCT, allowed us to fine tune the modelling of bias. In M7, we distinguish the bias due to selection into the study sample defined by equation (13) and the bias introduced possibly by differences between MSM who tested within the past 6 months and those not tested during this period, equation (12).

*Results.* Deviance estimates for model M7 are at an acceptable level both for the data informing the diagnosed and undiagnosed prevalence estimates (Table 2) and for the testing proportions (deviances not shown). Model M7 estimates a slightly lower number of diagnosed cases than model M3, 2071 *vs*. 2394, and a markedly lower number of undiagnosed cases, 3033 *vs*. 5063. In consequence the undiagnosed fraction in model M7 is lower, 60·0% (95% CrI 51·2–69·0), than in model M3, 68·3% (95% CrI 59·3–76·1), but the credible intervals largely overlap. Although the DIC value is larger for model M7, it is not directly comparable to the DIC for model M3, due to the differing and additional EMIS data included in the two models. We can, however, compare the contributions to the DIC resulting from the data that are common to the two models, namely the risk group proportion data, the diagnosis registry data and the VCT data. In model M3, these contributions sum to 34·9, whereas in model M7, they sum to 35·9. Since these contributions don't differ greatly, Model M7 may be considered an acceptable alternative to model M3, particularly as it provides additional information on testing patterns not available from the previous models.

### Regional and age differences: models M3, M7

**Fig. 4.**
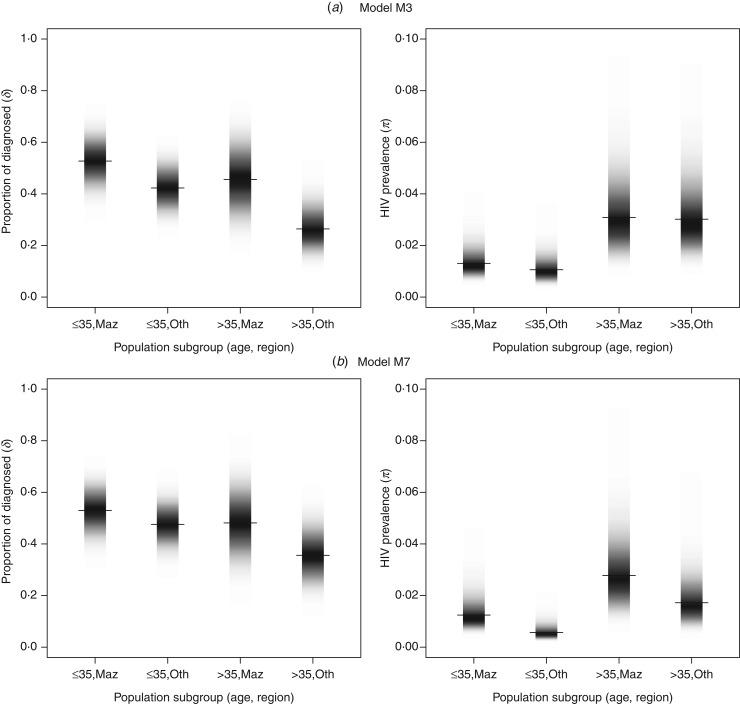
The prevalence and proportion of diagnosed infections in MSM by age and region in models M3 and M7 (darker colour represents higher density, median value is marked). (*a*) Model M3, (*b*) model M7.

Both models M3 and M7 demonstrate marked differences between regions and age groups (Fig. 4).

The prevalence is higher in older MSM. In model M3 the posterior probability that the prevalence is higher in MSM aged >35 years than in MSM aged ⩽35 years was *P* = 0·999 both in Mazowieckie and other regions. The respective posterior probability values for model M7 were 0·99 and 0·99. In the younger group, the prevalence tends to be higher in Mazowieckie than in other regions (M3: 1·3%, 95% CrI 0·8–2·3 *vs*. 1·0%, 95% CrI 0·8–1·8, posterior probability *P* = 0·92; M7: 1·2%, 95% CrI 0·7–2·4 *vs*. 0·6%, 95% CrI 0·3–1·1, posterior probability *P* = 0·99). The diagnosed fraction was the highest in younger MSM in Mazowieckie region, 52·7% (95% CrI 42·1–63·4) as estimated by model M3 and 53·0% (95% CrI 42·4–63·2) by M7. The posterior probabilities that the diagnosed fraction is higher in MSM in Mazowieckie aged ⩽35 years compared to MSM in Mazowieckie aged >35 years, and MSM in other regions aged ⩽35 and >35 years are 0·77, 0·92 and 0·99, respectively, in M3 and 0·70, 0·77 and 0·98, respectively, in M7.

Testing proportions could be estimated only from model M7. Past 6-month testing proportions were higher in the younger age group, especially in Mazowieckie, than in the older age group. Of MSM aged ⩽35 years in Mazowieckie (including the capital region) 32·6% (95% CrI 29·0–36·2) were tested in the previous 6 months compared to 23·1% (95% CrI 20·9–25·4) of MSM aged ⩽35 in other regions and 20·0% (95% CrI 14·0–27·0) of MSM aged >35 in Mazowieckie. The respective posterior probabilities that the proportion tested was higher in MSM aged ⩽35 years in Mazowieckie were both 0·99. The MSM aged >35, outside Mazowieckie region were the least likely to test (19·5%, 95% CrI 15·1–24·5). The posterior probabilities that the proportion was lower than in MSM aged ⩽35 in Mazowieckie, aged ⩽35 in other regions, and aged >35 in Mazowieckie were >0·999, 0·80 and 0·90, respectively.

## DISCUSSION

In this study, we investigated the possibility of estimating HIV prevalence and the fraction of infections that are undiagnosed in MSM in Poland, using existing evidence from diagnostic testing and behavioural surveys. Through analysing the conflicts between data and introducing bias parameters to the model, we were able to demonstrate that the available data possibly suffer from various biases. However, admitting bias in all data resulted in an inability to determine the main parameters of interest with sufficient precision. Only through imposing a significant constraint on the interrelation of biases were we able both to resolve the conflicts and to arrive at meaningful estimates.

Considering the family of models where the testing process was not explicitly modelled, the two models with satisfactory model fit criteria and good identifiabilty are: (1) the model allowing only the internet behavioural survey (EMIS) data to be biased (model M1); (2) the model constraining the biases in the EMIS and voluntary testing (VCT) data to be equal (model M3) (or, equivalently, drawn from the same distribution if the distribution has small variance as in model M4). However, these two models result in markedly different estimates of the key parameters of interest. The first one estimates the prevalence to be 9·9% (95% CrI 7·6–12·5) and the undiagnosed fraction to be 93·1% (95% CrI 88·9–95·9), whereas in the second model, the prevalence is 2·3% (95% CrI 1·4–3·9) and the undiagnosed fraction is 68·3·6% (95% CrI 59·3–76·1). The results of these two models could be understood as providing two different scenarios for the biases in the data, resolving conflicts between the data sources in two alternative ways. The first one assumes important bias in the EMIS data, while trusting that the assumed unbiased VCT data can resolve the conflict between the observed number of diagnoses and the population size, together with the prevalence of diagnosed infection, by reducing this latter quantity. However, in this scenario, prevalence is high and therefore the fraction of infections that are diagnosed is estimated to be very low. The second scenario, on the other hand, assumes that both the VCT and the EMIS data are similarily biased. This proposition is based on the assumption that the source population for both EMIS and VCT largely overlaps, indicating that MSM recruited by VCT and EMIS originate from the same sub-population of MSM, who may have a higher risk profile than the total MSM population in Poland. The conflict is then resolved by estimating lower prevalence to match the observed number of diagnoses.

Additional information would be necessary to differentiate between the two models, since they fit the data equally well according to the DIC. However, circumstantial evidence allows us to discuss the plausibility of the models. Crucially, the assumption that the VCT data might be unbiased is unrealistic. Self-referred testing is not usually a random process. Previous studies have found that testing may be related to risk behaviours. Those with higher risk practices present for testing more frequently than those with less risky behaviour, either due to perceived increased risk of infection or due to onset of symptoms of sexually transmitted infections [32–35]. Moreover, the estimated undiagnosed fraction of over 90% is questionable. While a considerable number of HIV-infected MSM present late, possibly indicating a large pool of undiagnosed infections, they constitute only ~10% of newly diagnosed cases [3]. Additionally, a significant proportion (at least 27%) of newly diagnosed cases in this group show evidence of recent infection [36], suggesting, on the contrary, frequent early diagnosis. These observations could be compatible with an early phase of an epidemic, when practically all infected individuals are at early stages of the disease. However, although HIV transmission between MSM in Poland appears to have increased in recent years, it has been documented since the late 1980s. Thus, if over 90% of infections were undiagnosed, we would expect a higher proportion of late-stage infections in the newly diagnosed population. Furthermore, in the respondents of the previous serosurvey of 2004, the undiagnosed fraction was 40% [12]. In the subsequent period, access to testing and testing promotion has increased: although no data are available to monitor the achieved coverage, a large increase of the undiagnosed fraction is unlikely. We therefore gave preference to the second model (model M3), with biases in VCT and EMIS data constrained to be the same. Similar assumptions (i.e. equality of biases) were inserted in model M7, in which more detailed data from the EMIS survey were included. Of note, the addition of these new data did not result in conflicting evidence, increasing confidence that the proposed relationships between the parameters are plausible.

Importantly both models with the bias constraints (M3 and M7), with lower undiagnosed fraction, indicate that an important number (5063, 95% CrI 3186-8124 in M3 and 3033, 95% CrI 1935–5099 in M7), or about 60–70% of MSM living with HIV in Poland may have in fact been undiagnosed in 2009. The estimated undiagnosed fraction is higher in older MSM and outside Mazowieckie (the capital region). In line with these estimates, we also demonstrate that the proportion tested in the past 6 months is highest in young MSM in Mazowieckie. These findings are consistent with the fact that the population reached by the VCT network tends to be young and living in cities. Testing options for MSM living outside of the main urban areas of Poland should be reviewed, taking into consideration the possible stigma associated with an HIV test in villages and small towns.

By contrast, the estimated overall prevalence is much lower than we might speculate based on diagnostic testing data or the EMIS self-reported HIV status data alone. The bias parameters (ORs) for both datasets, when constrained to be similar in M3, are estimated to be 6·5 (95% CrI 3·8–10·6), excluding 1, suggesting significant bias upwards in prevalence in both sources. The ORs estimated from M7 were even higher, 7·1 (95% CrI 3·7–12·5) in Mazowieckie and 11·6 (95% CrI 6·1–20·1) in other regions, also with credible intervals excluding 1. In terms of the EMIS survey, this is in line with what was observed across different participating countries: the respondents tended to be younger and reported HIV more frequently then expected based on other estimates [29]. Importantly, this suggests that both the internet channels used to recruit EMIS participants and the testing services attract the higher risk MSM subgroup. On one hand, this opens a window of opportunity to address prevention messages to those who need them the most, but on the other hand, this conclusion should stimulate discussion and design of interventions targeted towards individuals taking less risk, but still potentially infected and undiagnosed. These interventions may not necessarily have the same focus as the ones selected for the subgroup most at risk.

We note that there is little evidence of any difference between regions in the estimated prevalence for MSM aged >35 years (posterior probability that prevalence is higher in Mazowieckie is only *P* = 0·47 in M3 and *P* = 0·08 in M7). Although there are no behavioural data to explain such a finding, this is consistent with the surveillance data. The number of new HIV diagnoses in MSM was similar in Mazowieckie (including Warsaw) to other regions up to approximately 2002 [3]. It is plausible that the older age group was not as highly affected by outbreaks occurring in mid-2000, especially in the capital region, and thus the prevalence remains similar.

Our study was limited by the data availability and quality, forcing us to model additional uncertainty in almost all data sources included, via bias modelling. The sensitivity analysis led to the understanding that additional information (either as model constraints, or ideally, more and/or better quality data) is necessary in order to maintain good identifiability of the parameters. When relaxing the constraint on the biases in the model (increasing the parameter, *c*, determining the variance of the distribution of the biases) we notice that there exists a range of values of this parameter for which a transition is noticeable, between the two alternative scenarios.

Subsequently, and given the equal goodness of fit of the models, we favoured the model which gave more plausible results epidemiologically. We acknowledge that by imposing the constraint on the biases, we made a strong assumption on the interrelation of the data sources. Specifically, as discussed above, we assumed that data informing diagnosed and undiagnosed prevalence came from a similar population. Moreover, individual testing decisions may depend on multiple factors. In particular, those factors may depend on age, which would compromise our assumption that age differences of prevalence in the general MSM population are preserved also in the testers. The results should be therefore interpreted with caution.

Drawing robust conclusions regarding the epidemiological situation ideally requires collecting additional good quality data, including bio-behavioural surveys. The existing data sources could be explored for potential modelling of completeness of case-based surveillance data, in order to obtain a more precise estimate of the number diagnosed. Additionally, if more disaggregated behavioural data were to become available, the model could include a more comprehensive approach to bias adjustment, improving our understanding of the coverage of the data sources used.

This said, we conclude that even with imperfect and scarce data at hand, we were able to provide some new insights into the problem of HIV in the MSM population in Poland, by formally combining the available information and prior knowledge of data collection systems.

## Supplementary material

For supplementary material accompanying this paper visit http://dx.doi.org/10.1017/S0950268815002538. click here to view supplementary material
