# Supplementary material for: Bayesian evidence synthesis to estimate HIV prevalence in men who have sex with men in Poland at the end of 2009
Source: Epidemiol Infect. 2015 Nov 6;144(6):1175–91. doi: 10.1017/S0950268815002538 (PMC4825105; doi:10.1017/S0950268815002538)
Supplement: Supplementary file 1 [file S0950268815002538sup001.pdf]

Epidemiology and Infection

Bayesian evidence synthesis to estimate HIV prevalence among men who have sex with men in Poland at the end of 2009

M. Rosinska, P. Gwiazda, D. De Angelis, A.M. Presanis

Supplementary Material

1 Bayesian evidence synthesis

Bayesian inference is based on the factorisation of the posterior joint probability function, according to Bayes' theorem, into a prior distribution for the parameters $\boldsymbol{\theta}$ and the likelihood of the data $\boldsymbol{Y}$ given the parameters. The aim of evidence synthesis is to estimate k "basic" parameters $\boldsymbol{\theta} = \{\theta_1, \dots, \theta_k\}$ from n datasets $\boldsymbol{Y} = \{Y_1, \dots, Y_n\}$. The distribution of each dataset Y_i is determined by parameters that may be expressed as functions of the basic parameters: $Y_i \sim f(Y_i \mid G_i(\boldsymbol{\theta})) = f(Y_i \mid \boldsymbol{\theta})$. The function G_i is the identity function for a single component θ_j of $\boldsymbol{\theta}$, $G_i(\boldsymbol{\theta}) = \theta_j$ if the ith dataset directly informs θ_j. If the dataset indirectly informs multiple parameters, G_i is a more complex function of $\boldsymbol{\theta}$.

If each data source is assumed independent, a likelihood function combining all data items may be constructed as the product of contributions from each data source, $L(\boldsymbol{Y} \mid \boldsymbol{\theta}) = \prod_{i=1}^{n} L(Y_i \mid \boldsymbol{\theta})$.

Additionally, other pre-existing knowledge may be added in the form of the prior distribution on $\boldsymbol{\theta}$, determined by additional *hyper-parameters* $\boldsymbol{\lambda}$: $f(\boldsymbol{\theta} \mid \boldsymbol{\lambda})$. Using the Bayesian approach, the joint posterior distribution of $\boldsymbol{\theta}$ conditional on the available data and hyper-parameters is:

$$f(\boldsymbol{\theta} \mid \boldsymbol{Y}, \boldsymbol{\lambda}) = \frac{f(\boldsymbol{Y} \mid \boldsymbol{\theta}) \times f(\boldsymbol{\theta} \mid \boldsymbol{\lambda})}{f(\boldsymbol{Y} \mid \boldsymbol{\lambda})} \propto f(\boldsymbol{Y} \mid \boldsymbol{\theta}) \times f(\boldsymbol{\theta} \mid \boldsymbol{\lambda}) = L(\mathbf{Y} \mid \boldsymbol{\theta}) \times f(\boldsymbol{\theta} \mid \boldsymbol{\lambda})$$

The normalising factor $\frac{1}{f(\boldsymbol{Y} \mid \boldsymbol{\lambda})}$ does not depend on the parameters $\boldsymbol{\theta}$ for which we want to find the posterior distribution. However, it may not be straight forward to evaluate. For this reason, the joint posterior distribution for the basic parameters, as well as their marginal distributions, are estimated using a Monte Carlo Markov Chain algorithm in

the OpenBUGS software [2]. In this method, the joint posterior distribution is the steady state distribution of a Markov Chain, defined by an iterative sampling procedure, which uses information on the relative probability of certain states derived from $L(\boldsymbol{Y} \mid \boldsymbol{\theta}) \times f(\boldsymbol{\theta} \mid \boldsymbol{\lambda})$.

2 Construction of the likelihood and the joint posterior distribution function - initial model

In our model, the basic parameters of interest in $\boldsymbol{\theta}$ are: ν, the proportion of MSM among males; $\pi_{age,reg}$, HIV prevalence by age and region; and $\delta_{age,reg}$, the proportion diagnosed among HIV positives by age and region.

Contributions from data informing proportions The data available that inform proportions are: the number of MSM, y^{pop}, among men, n^{pop}; the number of previously undiagnosed HIV-positive MSM, $y^{vct}_{age,reg}$, by age and region, among MSM undertaking VCT, $n^{vct}_{age,reg}$; and the number of diagnosed HIV-positive MSM, $y^{emi}_{age,reg}$, among MSM in the EMIS survey, $n^{emi}_{age,reg}$, by age. Each numerator y is assumed to be a realisation of a Binomial distribution, specified in terms of the functions $G_i = G_i(\nu, \pi_{age,reg}, \delta_{age,reg})$ as follows:

$$
\begin{aligned}
y^{pop} &\sim Binomial(n^{pop}, G^{pop}), & G^{pop} &= \nu \\
y^{vct}_{age,reg} &\sim Binomial(n^{vct}_{age,reg}, G^{vct}_{age,reg}), & G^{vct}_{age,reg} &= u_{age,reg} = \pi_{age,reg}(1 - \delta_{age,reg}) \\
y^{emi}_{age,reg} &\sim Binomial(n^{emi}_{age,reg}, G^{emi}_{age,reg}), & G^{emi}_{age,reg} &= dp_{age,reg} = \pi_{age,reg}\delta_{age,reg}
\end{aligned}
$$

The likelihood contribution of each data item coming from a binomial distribution is defined as:

$$L(Y_i \mid \nu, \pi, \delta) = \binom{N_i}{Y_i} \cdot G_i^{Y_i} \cdot (1 - G_i)^{N_i - Y_i}$$

Contributions from count data Next, we have data on the number of reported cases, which we assume to be Poisson distributed with the mean $\mu_{age,reg}$:

$$y^{rep}_{age,reg} \sim Poisson(G^{rep}_{age,reg}), \qquad G^{rep}_{age,reg} = \mu^{rep}_{age,reg}$$

$$y^{unk}_{age,reg} \sim Poisson(G^{unk}_{age,reg}), \qquad G^{unk}_{age,reg} = \mu^{unk}_{age,reg}.$$

The likelihood contributions for each data point representing a realisation from Poisson distribution are:

$$L(Y_i \mid \nu, \pi, \delta) = \frac{e^{-G_i} \cdot G_i^{Y_i}}{Y_i!}$$

We assume that the true number of diagnosed cases lies between the number of newly diagnosed HIV cases reported as MSM and the total of male HIV cases reported as MSM and those reported with missing transmission category:

$$\mu^{rep}_{age,reg} \leq d_{age,reg} \leq \mu^{rep}_{age,reg} + \mu^{unk}_{age,reg},$$

We consider parameters μ as additional basic parameters and assign them flat prior distributions:

$$\mu^{rep}_{age,reg} \sim Gamma(0.01, 0.01)$$

$$\mu^{unk}_{age,reg} \sim Gamma(0.01, 0.01)$$

In order to ensure that the above inequalities hold, we also introduce additional parameters $\alpha_{age,reg}$ and artificial data points $a_{age,reg}$, which are realisations from Bernoulli distributions with success probability $\alpha_{age,reg}$:

$$a_{age,reg} \sim Bernoulli(\alpha_{age,reg})$$

The parameters $\alpha_{age,reg}$ are defined as functions of other parameters as follows:

$$\alpha_{age,reg} = \begin{cases} 1 & \text{if } \mu^{rep}_{age,reg} \leq d_{age,reg} \leq \mu^{rep}_{age,reg} + \mu^{unk}_{age,reg} \\ 0 & \text{otherwise} \end{cases}$$

We consider that the above inequalities hold and this belief is included in the model as data points informing the Bernoulli distribution, i.e.:

$$a_{age,reg} = 1.$$

The likelihood function The complete formulation of the likelihood function becomes:

$$
\begin{aligned}
L(\mathbf{Y} \mid \nu, \pi, \delta, \mu) &= \prod_{i=1}^{n} L(Y_i \mid \nu, \pi, \delta, \mu) \\
&= \binom{n^{pop}}{y^{pop}} \cdot \nu^{y^{pop}} \cdot (1-\nu)^{(n^{pop}-y^{pop})} \cdot \\
&\prod_{age}\Bigg\{\prod_{reg}\Bigg\{\binom{n^{emi}_{age,reg}}{y^{emi}_{age,reg}} \cdot (dp_{age,reg})^{y^{emi}_{age,reg}} \cdot (1-dp_{age,reg})^{(n^{emi}_{age,reg}-y^{emi}_{age,reg})} \cdot \\
&\binom{n^{vct}_{age,reg}}{y^{vct}_{age,reg}} \cdot (u_{age,reg})^{y^{vct}_{age,reg}} \cdot (1-u_{age,reg})^{(n^{vct}_{age,reg}-y^{vct}_{age,reg})} \cdot \\
&\frac{e^{-\mu^{rep}_{age,reg}} \cdot (\mu^{rep}_{age,reg})^{(y^{rep}_{age,reg})}}{(y^{rep}_{age,reg})!} \cdot \frac{e^{-\mu^{unk}_{age,reg}} \cdot (\mu^{unk}_{age,reg})^{(y^{unk}_{age,reg})}}{(y^{unk}_{age,reg})!} \cdot \\
&(\alpha_{age,reg})^{a_{age,reg}} \cdot (1-\alpha_{age,reg})^{(1-a_{age,reg})}\Bigg\}\Bigg\}.
\end{aligned}
$$

Prior distribution The parameters determining binomial distributions were given vague priors (uniform distributions $Uniform[\lambda_1, \lambda_2]$, where $\lambda_1 = 0$ and $\lambda_2 = 1$), and the parameters μ were assumed to have vague $Gamma(\lambda_3, \lambda_4)$ distributions, where $\lambda_3 = \lambda_4 = 0.01$:

$$
f(\nu, \pi, \delta \mid \lambda) = \begin{cases} A & \text{if } \nu, \pi_{age,reg}, \delta_{age,reg} \in [0,1] \; and \; \mu^{rep}_{age,reg}, \mu^{unk}_{age,reg} > 0 \\ 0 & \text{otherwise} \end{cases}
$$

where $A = (\frac{(0.01)^{(0.01)}}{\Gamma(0.01)})^4 \cdot \prod_{age,reg}(\mu^{rep}_{age,reg} \cdot \mu^{unk}_{age,reg})^{(0.99)} \cdot e^{-0.01\cdot(\mu^{rep}_{age,reg}+\mu^{unk}_{age,reg})}$ denotes the gamma distributed priors.

3 Model criticism using deviance summaries

Deviance summaries were used to evaluate the fit of particular models and to discriminate between the models. For the parameter vector θ and data y, the deviance function $D(\theta, y)$ is defined as follows:

$$
D(\theta, y) = -2log(p(y \mid \theta)) + 2log(h(y))
$$

where the function $h(y)$ does not depend on θ, and is of the form $p(y \mid \hat{\theta})$. We will assume that $\hat{\theta}$ is the maximum likelihood estimator (MLE) of θ calculated under the

assumption of a saturated model (i.e. independently for each data item). In our model, for the data coming from binomial distributions, $\hat{\theta}$ is the observed proportion and for the data from the Poisson distributions, the MLE is the observed count.

Given the posterior distribution of θ, $p(\theta|y)$, the posterior expected deviance $\overline{D}(y)$ is defined as:

$$\overline{D}(y) = E_{\theta|y} D(\theta, y) = \int D(\theta, y) p(\theta|y) d\theta.$$

The posterior expected deviance can be evaluated through the MCMC procedure. Once the chain has converged we expect the values of the parameters θ generated at each step to come from the posterior distribution of θ. $D(\theta, y)$ is then calculated at each interation for the generated values of θ and the $\overline{D}(y)$ can be approximated by the mean value of the values calculated at each iteration.

Under the independence assumption for each data item, we may examine $\overline{D}_i(y)$, the contribution of Y_i to the overall posterior mean deviance. If the proposed model fits well the data item Y_i, then $\overline{D}_i(y) \approx 1$ [1, 3]. If the assumed model is true, then $\overline{D}(y) = \sum_i \overline{D}_i(y)$ is approximately equal to the number of parameters in the saturated model.

The model complexity is defined by the effective number of parameters $p_D = \overline{D}(y) - D(\overline{\theta}, y)$, where $\overline{\theta}$ is the posterior mean of θ. Finally, to discriminate between different models, we used the Deviance Information Criterion (DIC) defined as:

$$DIC = D(\overline{\theta}, y) + 2p_D = \overline{D}(y) + p_D.$$

The model with the smallest DIC is generally the preferred one, optimising the fit with the least complexity.

4 Trends in (undiagnosed) prevalence - VCT data

Following Section 2.3 of the main text, we assume that the undiagnosed prevalence evolved during 2010 depending on the rate of new infections r_i and the rate of diagnoses r_d as follows:

$$u(t) = u(t_0) + (r_i - r_d)(t - t_0) \quad (1)$$

where $t \in [t_0, t_1]$, t_0 denotes 01.01.2010, t_1 is 31.12.2010 and both r_i and r_d were constant during this time. We also assume that the overall population of MSM is stable over the course of the year, i.e. that entries and exits to the population (due to aging, migration, death and behaviour change) cancel.

Further, we assume that the prevalence among MSM testing at the VCT network at a given time point approximates the undiagnosed prevalence. As the date of test (month) is available for 99.8% of the testers, we are in a position to study the dependence of prevalence on the month of test in the VCT data. The general trend is displayed in Figure 1. We note a small downward tendency. However, when running a binomial regression model adjusting for age and region, this trend is not statistically significant (Table 1). In this model, we used the identity link to better test the assumption of a linear effect of time on undiagnosed prevalence. The logit link is more common when modelling a proportion, but in our case, since we consider a short time period (1 year) and given the order of magnitude of the outcome (proportion positive), the model is unlikely to predict values of the proportion outside the $[0, 1]$ range.

Figure 1: Trend in undiagnosed prevalence, u(t), observed in VCT testers during 2010

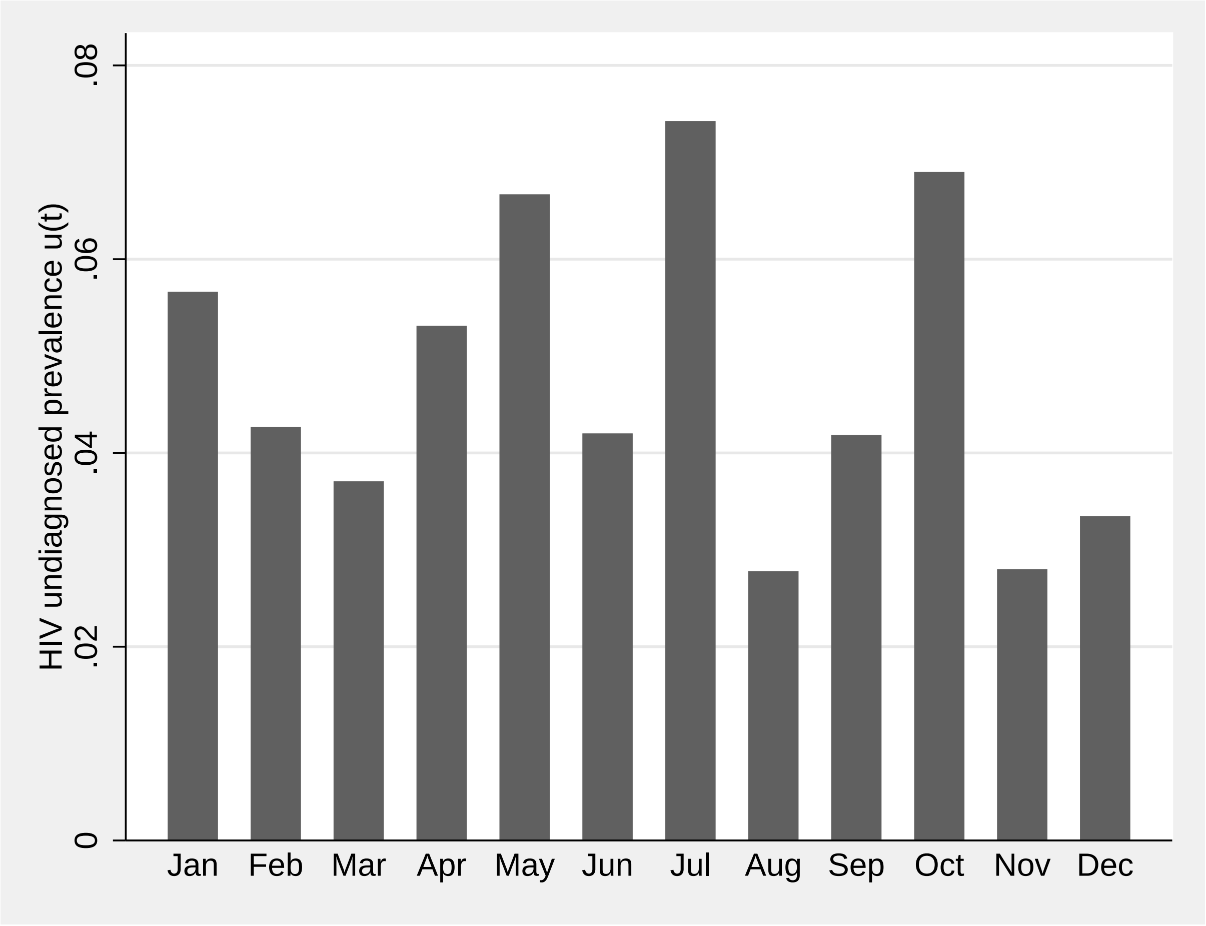

5 EMIS and VCT study populations

The characteristics of the subpopulations of MSM recruited in EMIS survey and the ones volunteering for testing in VCT are provided in Table 2.

Table 1: Binomial regression model of HIV positive result on time of test, region and age group in VCT data

	Risk difference	Std.Err.	z	p>z	[95% Conf. Int.]	
Time of the test (linear effect, per 1 additional year)	-0.0099	0.01287	-0.77	0.441	-0.035	0.015
Region (Oth vs Maz)	0.0101	0.0074	1.37	0.171	-0.004	0.025
Age (> 35 vs ≤ 35)	0.0715	0.0166	4.31	0.000	0.039	0.104
Constant	0.0380	0.0091	4.19	0.000	0.0202	0.0558

References

[1] **Dempster AP**. The direct use of likelihood for significance testing. *Statistics and Computing* 1997; **7**: 247-252. http://dx.doi.org/10.1023/a:1018598421607. Accessed 24 October 2014.

[2] **Lunn D, *et al*.** The BUGS project: Evolution, critique and future directions. *Statistics in Medicine* 2009; **28**: 3049-3067. http://dx.doi.org/10.1002/sim.3680. Accessed 24 October 2014.

[3] **Spiegelhalter DJ, *et al*.** Bayesian measures of model complexity and fit (with discussion). *Journal of the Royal Statistical Society: Series B* 2002; **64**: 583–639.

Table 2: Comparison of the characteristics of the EMIS respondents and VCT clients

		EMIS		VCT	
		N	%	N	%
Age (years)	15 – 24	896	30.17	1034	37.85
	25 – 39	1663	55.99	1514	55.42
	40+	311	10.47	184	6.73
Education	Elementary	82	2.86	162	5.66
	Vocational	99	3.45	167	5.83
	High school	1182	41.18	1152	40.24
	Higher	1484	51.71	1233	43.07
	N.D.	23	0.80	149	5.20
Region	Dolnoslaskie	247	8.61	204	7.13
	Kujawsko-pomorskie	105	3.66	85	2.97
	Lubelskie	44	1.53	50	1.75
	Lubuskie	35	1.22	48	1.68
	Lodzkie	123	4.29	67	2.34
	Malopolskie	285	9.93	90	3.14
	Mazowieckie	972	33.87	1295	45.23
	Opolskie	29	1.01	34	1.19
	Podkarpackie	25	0.87	21	0.73
	Podlaskie	23	0.80	56	1.96
	Pomorskie	154	5.37	196	6.85
	Slaskie	206	7.18	197	6.88
	Swietokrzyskie	21	0.73	36	1.26
	Warminsko-mazurskie	32	1.11	54	1.89
	Wielkopolskie	225	7.84	232	8.10
	Zachodniopomorskie	90	3.14	144	5.03
	N.D.	254	8.85	54	1.89
Prior HIV test	Never tested	1057	36.83	-	
	Tested, positive	147	5.12	-	
	Tested, last test negative	1649	57.46	-	
	N.D.	17	0.59	-	
Time of last HIV test (among not previously diagnosed)	Never tested	1057	39.06	1245	43.49
	past 12M	960	35.48	849	29.65
	>12M	394	14.56	633	22.11
	N.D.	295	10.90	136	4.75
Location of last HIV test	VCT	802	44.65	980	61.60
	other	993	55.29	271	17.03
	N.D.	1	0.06	340	21.37
Number of casual sex partners past 12 M	None	783	27.28	469	16.38
	1-5	1152	40.14	1383	48.31
	> 5	813	28.33	470	16.42
	N.D.	122	4.25	541	18.90
